# Cell-Penetrating Penta-Peptides (CPP5s): Measurement of Cell Entry and Protein-Transduction Activity

**DOI:** 10.3390/ph3123594

**Published:** 2010-12-15

**Authors:** Jose A. Gomez, Joseph Chen, Justine Ngo, Dagmar Hajkova, I-Ju Yeh, Vivian Gama, Masaru Miyagi, Shigemi Matsuyama

**Affiliations:** 1 Department of Medicine, Division of Hematology/Oncology, Case Western Reserve University, Cleveland, OH 44106, USA; 2 Department of Pharmacology, Case Western Reserve University, Cleveland, OH 44106, USA; 3 Case Center for Proteomics and Bioinformatics, Case Western Reserve University, Cleveland, OH 44106, USA; 4 Department of Ophthalmology, Vision Science Research Center, Case Western Reserve University, Cleveland, OH 44106, USA; 5 Division of General Medical Science, Oncology Case Comprehensive Cancer Center, Cleveland, OH 44106, USA

**Keywords:** Cell-Penetrating Peptide (CPP), Cell-Penetrating Penta-peptide (CPP5), Bax Inhibiting Peptide (BIP), Protein Transduction, Ku70, Bax, Apoptosis, Drug Delivery

## Abstract

Previously, we developed cell-penetrating penta-peptides (CPP5s). In the present study, VPTLK and KLPVM, two representative CPP5s, were used to characterize the cell-penetration and protein-transduction activities of these small molecules. Various inhibitors of endocytosis and pinocytosis (chlorpromazine, cytochalasin D, Filipin III, amiloride, methyl-β-cyclodextrin, and nocodazole) were tested. Only cytochalasin D showed suppression of CPP5 entry, though the effect was partial. In addition, CPP5s were able to enter a proteoglycan-deficient CHO cell line. These results suggest that pinocytosis and endocytosis may play only a minor role in the cell entry of CPP5s. By mass spectrometry, we determined that the intracellular concentration of VPTLK ranged from 20 nM to 6.0 μM when the cells were cultured in medium containing 1 μM – 1.6 mM VPTLK. To determine the protein-transduction activity of CPP5s, the Tex-LoxP EG cell line, which has a Cre-inducible green fluorescent protein (GFP) gene, was used. VPTLK and KLPVM were added to the N-terminus of Cre, and these fusion proteins were added to the culture medium of Tex-LoxP EG cells. Both VPTLK-Cre and KLPVM-Cre were able to turn on GFP expression in these cells, suggesting that CPP5s have protein-transduction activity. Since CPP5s have very low cytotoxic activity, even at a concentration of 1.6 mM in the medium, CPP5s could be utilized as a new tool for drug delivery into cells.

## 1. Introduction

Cell-penetrating peptides (CPPs) comprise a group of peptides that is able to penetrate the plasma membrane of living cells [1,2,3,4,5,6]. The development of the CPPs began following the discovery that the Human Immunodeficiency Virus (HIV) TAT transactivator protein was able to enter human cells [7,8]. Based on this discovery, cell-penetrating TAT peptide [10,11,12,13,14 amino acids (a.a.)] was developed [9,10,11]. Later, it was reported that the homeobox domain (α-helix) of the antennapedia protein of *Drosophila* was able to penetrate into mammalian cells; the penetratin peptide (16 a.a.) was designed based on the homeobox domain of this protein [12,13]. In addition to TAT and penetratin, several new CPPs have been discovered, including synthetic arginine-rich peptides (8 a.a.) [5,14,15], amphipathic peptides (18 a.a.) [1,16], and transportan (21 a.a.) [1,17,18], among others [19,20,21]. These CPPs have also been reported to show protein-transduction activity; that is, the ability to carry large cargo proteins from the culture medium into the intracellular space [19,20,21].

**Figure 1:**
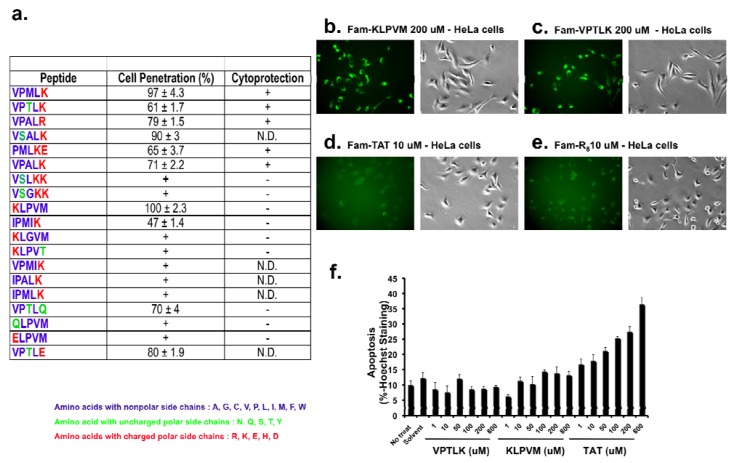
Cell-penetration activity of CPP5s. *a.* List of cell-penetrating penta-peptides (CPP5s). Left column shows the amino acid sequence. Peptide sequences are color-coded to show the constant amphipathic amino acid arrangement in each CPP5. Cell-penetration activity was determined by fluorescence intensity of cells (DAMI cells: a human megakaryocytic cancer cell line) incubated with fluorescein (Fam)-labeled peptides (200 μM) for 3 h at 37 °C as described in Experimental Procedures. Fam was labeled on the N-terminus of all peptides examined. Cells were analyzed by FACS and/or microscopy. The activity of each peptide is shown as a percentage of the activity of KLPVM. Each value represents the mean of triplicate samples. Cell entry of some peptides was confirmed by microscopy only, and these peptides are indicated with a ‘+’ without a percentage value. For cytoprotective activity (right column), the information is based on previous studies [6,22,23,25,27,28] and the activity was determined by the protection of cells from Bax overexpression and other apoptotic stimuli such as etoposide treatment. *b to e.* Cell entry of Fam-labeled VPTLK, KLPVM, TAT, and poly-arginine (R_8_). HeLa cells were incubated with Fam-labeled VPTLK, KLPVM (200 μM), Fam-TAT (10 μM), and Fam-R_8_ (10 μM) for 1 h at 37 °C. Images are at 20× magnification. *f.* Comparison of the cytotoxicity of CPP5 and TAT. Primary cultured mouse embryonic fibroblast (MEF) cells were treated with various concentrations of peptides for 24 h as indicated. Then, apoptosis was analyzed as described in Experimental Procedures. Each bar represents the mean of triplicate samples and standard error.

The cell-penetrating penta-peptides (CPP5s) derived from the protein Ku70 were initially designed based on the Bax-binding domain of Ku70 [6,22,23]. Ku70 is a multifunctional protein involved in non-homologous end-joining DNA repair and cell-death regulation (reviewed in [24]). Ku70 binds and inhibits the pro-apoptotic protein Bax in the cytosol, thereby inhibiting Bax activation and translocation from the cytosol to the mitochondria [6,22,23,25]. A series of cell-penetrating anti-apoptotic peptides was designed based on the Bax-binding domain of Ku70 of various species, including human, rat, and mouse [6,23]. In addition, other CPP5s were developed by scrambling the sequence. These latter, non-cytoprotective CPP5s were originally intended to serve as negative-control peptides to verify the cytoprotective activity of the Ku70-derived CPP5s. The amino acid sequences of these CPP5s are shown in Figure 1a. 

The mechanism of cell penetration exerted by CPPs is not yet understood; however, the sequences of the peptides hint at possible mechanisms. For example, the positively charged amino acids of TAT and poly-arginine may facilitate peptide interaction with negatively charged receptor proteins (e.g. proteoglycan) [26] or charged phospholipids at the surface of cells [20]. These interactions are hypothesized to trigger endocytosis or macropinocytosis of CPPs. However, it is still unclear how CPPs in the enodsome can pass through the endosomal membrane to enter the cytosol [19].

In this study, to quantify the cell-penetrating activity of CPP5s, we measured the intracellular concentration of CPP5s by using mass spectrometry. Furthermore, the protein-transduction activity of CPP5s was determined by measuring the ability of CPP5s to deliver Cre DNA recombinase (as fusion proteins) into cells. The results show that an intracellular CPP5 concentration of 6 µM can be reached without significant toxicity, and that CPP5s have protein-transduction activity. Thus CPP5s could be potentially utilized as tools to deliver therapeutic molecules into cells.

## 2. Results and Discussion

### 2.1. List of CPP5s

The amino acid sequences of characterized CPP5s are shown in Figure 1a. CPP5s that were designed based on the Bax-binding domain of Ku70 of several species are named Bax-Inhibiting Peptides (BIPs); they are VPMLK, PMLKE, VPTLK, VPALR, and VPALK. VPMLK and PMLKE were designed from human Ku70, and the same sequence exists in the Ku70 homologues of dog and monkey. VPTLK and VPALR are based on mouse and rat Ku70, respectively. VPALK was designed from cattle Ku70, and the same sequence exists in Ku70 of the African clawed frog. Cytoprotective activities of these BIPs have been previously reported, both in cell culture and animal models [6,22,23,25,27,28]. Among the CPP5s, KLPVM was found to have the best cell-penetrating activity when assessed by flow cytometric analysis of the cellular fluorescence intensity of Fam (5,6-carboxyfluorescein)-labeled CPP5s (Figure 1a). The observed variation in cell-entering activity of Fam-labeled-CPP5s is shown in Figure 1a as a percentage of the activity of KLPVM. In all cases, Fam was attached to the N-terminal amino acid. For some CPP5s, cell-penetration activity was confirmed by detection of an intracellular fluorescent signal by microscopy. These latter results are indicated with ‘+’ symbols, and a quantitative comparison with the activity of KLPVM was not performed. Representative images of the cell entry of Fam-labeled KLPVM and VPTLK are shown in Figure 1b and c. As positive controls, cells cultured in the presence of Fam-TAT and Fam-octa-arginine (R8) are also shown (Figure 1d and Figure 1e). As previously reported [6,29], Fam-labeled CPP5s showed detectable cell-penetrating activity when the peptides were added to the culture medium at concentrations greater than 1 µM. In this study, we used KLPVM and VPTLK as representative CPP5s for further analysis of the cell-penetrating activity. 

### 2.2. Cytotoxicity analysis

The cytotoxicities of CPP5s and TAT were compared in mouse embryonic fibroblasts (MEFs). MEFs were cultured for 24 h in the presence of various concentrations of VPTLK, KLPVM, and TAT, as indicated in Figure 1f. VPTLK and KLPVM did not show significant cytotoxic effects even at 1.6 mM in the cell culture medium. In contrast, TAT showed apoptosis-inducing activity when its concentration in the medium rose above 100 μM.

### 2.3. Mechanism of cell entry of CPP5s

**Figure 2:**
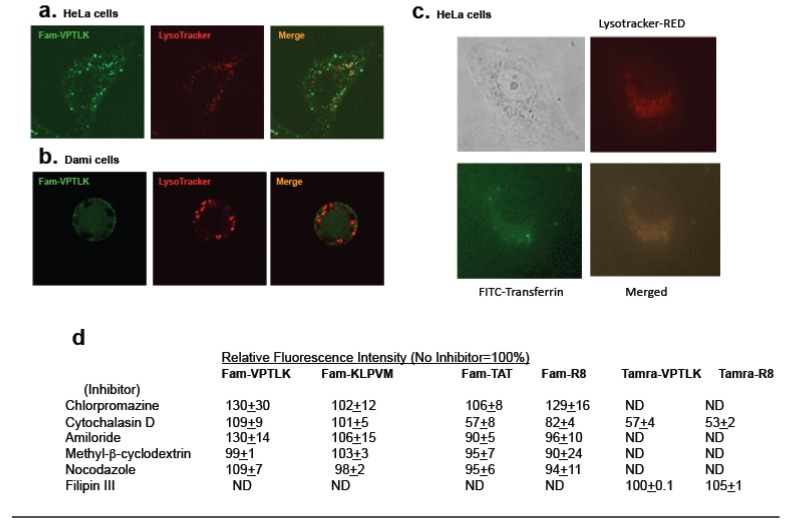
Mechanism of cell entry of CPP5s. *a* and *b.* VPTLK partially co-localizes with lysosomes. Microscopic analysis of the co-localization of Fam-VPTLK with lysosome-staining dye (LysoTracker™ red DND-99, L7528 Invitrogen). Hela cells (*a)* and DAMI cells (*b)* were incubated with Fam-VPTLK (50 μM) and LysoTracker (100 nM) for 1 h at 37 °C. *c.* FITC-labeled Transferin (FITC-Transferin) co-localized with lysosome. HeLa cells were incubaled with FITC-transferin (100 nM) and LysoTracker (100 nM) for 1 h at 37 °C. *d.* Effects of inhibitors of pinocytosis and endocytosis on the cell-penetrating activity of CPP5s. HeLa cells were pre-treated with various pinocytosis inhibitors for 30 min. Then, Fam- or Tamra-labeled peptides were added to the culture medium. Cells were incubated with peptides for 1 h and the cellular fluorescence intensity was measured by fluorescence microscopy equipped with image analysis software (for Fam-labeled peptides) or FACS (for Tamra-labeled peptides) as described in Experimental Procedures. VPTLK and KLPVM were used at 30 μM and TAT and RRRRRRRR (R8) were used at 10 μM. The results are shown as percentages of the fluorescence intensity of inhibitor-treated cells (the intensity of non-treated cells is designated as 100%). Each value represents mean of three experiments and standard error. The pinocytosis inhibitors used were: Chlorpromazine (an inhibitor of chlathrin-mediated endocytosis), cytochalasin D (an inhibitor of F-actin elongation, resulting in inhibition of macropinocytosis and caveolin-mediated endocytosis), amiloride (an inhibitor of macropinocytosis), methyl beta cyclodextrin (it depletes cholesterol from the plasma membrane, thus inhibiting cell entry dependent on lipid rafts), nocodazole (an inhibitor of microtubule formation), and Filipin III (an inhibitor of macropinocytosis).

Lysosomes are known to contain molecules which have entered the cell through pinocytosis [30]. To determine whether CPP5s use pinocytotic pathways for their cell entry, the localizations of Fam-labeled CPP5s and lysosomes (labeled by LysoTracker® Red) were compared. Only a subset of Fam-VPTLK peptides co-localized with lysosomes while the majority did not (Figure 2a and b). In contrast, FITC-labeled transferrin and LysoTracker-Red co-localized extensively, which is consistent with the understanding that transferrin enters cells through receptor-mediated endocytosis (Figure 2c). Partial co-localization of Fam-VPTLK with LysoTracker suggests that the cell penetration of CPP5s is achieved through both pinocytosis-dependent and -independent pathways.

To further examine the role of pinocytosis, various pinocytosis inhibitors were tested as shown in Figure 2d. HeLa cells were incubated with each inhibitor in serum-free DMEM for 30 min, followed by incubation for 1 h with Fam-labeled CPP5 peptides (30 μM). Then, the cells were extensively washed with Hanks’ Buffered Salt Solution (HBSS) and complete DMEM, prior to the detection of fluorescence by microscopy and FACS. None of amiloride, chlorpromazine, cytochalasin D, Filipin III, nocodazole and methyl-beta-cyclodextrin blocked the entry of Fam-labeled VPTLK and KLPVM into HeLa cells. However, when Tamra-labeled VPTLK was used, cytochalasin D significantly attenuated the cell entry of VPTLK (57±4% of the non-treated control). As a control experiment to validate the effectiveness of these inhibitors, FITC-transferrin (for chlorpromazine) and rhodamine-labeled EGF (for cytochalasin D and amiloride) were used. These inhibitors significantly suppressed the cell entry of transferrin and EGF, as HeLa cells were unable to take up detectable FITC-transferrin and rhodamine-EGF (not shown). To compare the cell-penetrating mechanism between CPP5s and arginine-rich CPPs, the uptake of TAT and R8 was also examined in the presence of these inhibitors. Only cytochalasin D had a suppressive effect on the cell-penetrating activity of TAT and R8, consistent with a previous report [31].

**Figure 3:**
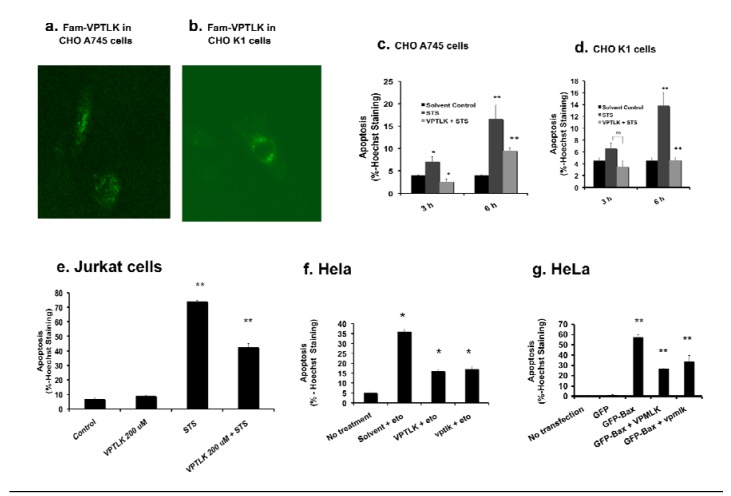
Protection of cells from apoptosis by VPTLK. *a-d.* Proteoglycan is not required for VPTLK cell entry. Microscopic analysis of cell entry of Fam-VPTLK in Chinese Hamster Ovary (CHO) A745 cells lacking proteoglycans in the plasma membrane (*a)* and CHO K1 cells that express proteoglycans (*b)*. CHO A745 and K1 cells were incubated with Fam-VPTLK (200 μM) for 3 h at 37 °C. VPTLK (3 h pre-incubation) protected both cell lines from 100 nM staurosporine (STS) treatment (3, and 6 h) (*c and d)*. Apoptosis was determined by using Hoechst dye nuclear staining. Each bar represents the mean of triplicate samples and standard error, and statistical significance was determined by an unpaired student *t* test; “ns”: not significant, *P < 0.05, and **P < 0.01. *e*. VPTLK protected Jurkat cells from STS-induced apoptosis. Jurkat cells were pre-incubated with VPTLK 200 μM for 3 h prior to STS treatment (100 nM, 3 or 6 h). Apoptosis was determined by Hoechst dye nuclear staining. Each bar represents the mean of triplicate samples and standard error, and statistical significance was determined by an unpaired student *t* test: **P < 0.01. *f.* VPTLK and D-type VPTLK (vptlk^D^) protected HeLa cells from etoposide-induced apoptosis. HeLa cells were pretreated with 200 μM VPTLK or vptlk^D^ for 3 h prior to etoposide treatment (10 μM, 24 h). Apoptosis was determined by Hoechst dye nuclear staining. Each bar represents the mean of triplicate samples and standard error. ** P < 0.01 *g.* VPTLK and vptlk^D^ inhibited Bax-induced apoptosis in HeLa cells. HeLa cells were pretreated with VPTLK and vptlk^D^ 200 μM for 3 h prior to pEGFP-Bax plasmid transfection. Apoptosis was assayed 24 h after the transfection as described in Experimental Procedures. Each bar represents the mean of triplicate samples and standard error. ** P < 0.01

Chinese hamster ovary (CHO) A745 cells lack proteoglycans in the plasma membrane [32], and this cell line has been used to determine whether proteoglycans are required for CPP entry [32]. In CHO A745, as well as in its parental cell line CHO K1 that expresses proteoglycans, Fam-VPTLK and Fam-KLPVM (not shown) both showed cell-penetrating activity (Figure 3a and b), and VPTLK showed cytoprotective activity [*i.e*., VPTLK protected these cells from the apoptosis-inducing chemical staurosporine (STS)] (Figure 3c and d). The cell-penetrating activity of CPP5s was also examined in Jurkat cells that are reported to lack caveolin-mediated endocytotic activity [33]. Fam-VPTLK and -KLPVM were able to enter Jurkat cells (not shown) and VPTLK showed cytoprotective activity in this cell type (Figure 3e). These results suggest that CPP5s do not require proteoglycan- and caveolin-mediated endocytosis for their cell-penetrating activity.

Previously, D-type amino acid-based peptides were shown to have better stability inside cells because they are resistant to proteases [34]. Therefore, we expected that D-type VPTLK might show better cytoprotective activity because of its higher intracellular stability. Interestingly, both L-type and D-type VPTLK showed similar cytoprotective activity against etoposide treatment and Bax overexpression (Figure 3f and g). These results suggest that the cell-entry pathway of CPP5s is not selective for L- or D-type amino acids. 

### 2.4. Quantification of the cell-penetrating activity of VPTLK by mass spectrometric analysis

**Figure 4:**
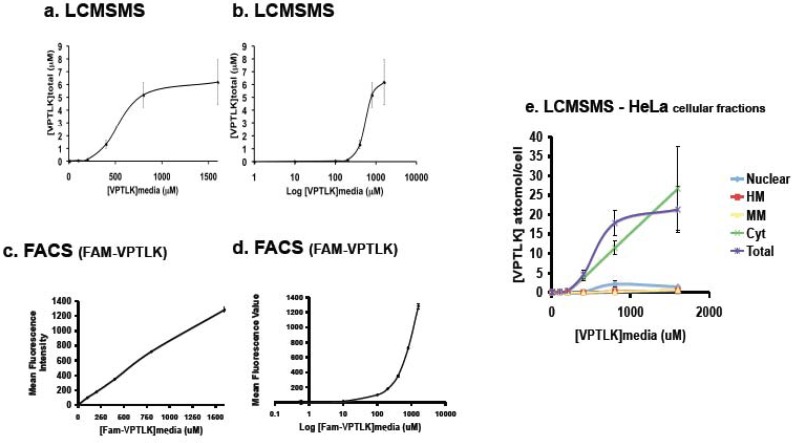
Quantification of VPTLK concentration in HeLa cells by mass spectrometry (MS). *a* and *b.* VPTLK concentration in HeLa cells (y-axis) incubated in various concentrations of VPTLK in the culture medium (x-axis). HeLa cells were incubated for 24 h in VPTLK-containing medium. Then the cells were collected and the VPTLK concentration was measured using MS as described in the Experimental Procedures. Each value represents the mean of triplicate samples and standard error. *b.* Semi-Logarithmic presentation of the data in *(a)*. *c* and *d.* Fluorescence-Activated Cell Sorting (FACS) analysis of the cell penetration of Fam-VPTLK into HeLa cells. Cells were incubated for 3 h in various concentrations of VPTLK in the medium. Each value represents the mean of triplicate samples and standard error. *d.* Semi-Logarithmic profile of the data shown in *(c)*. *e.* VPTLK concentration in each subcellular fraction of HeLa cells cultured in various concentrations of VPTLK. The graph shows the VPTLK concentration in the cytolsolic fraction (Cyt), heavy membrane (HM), nucleus, or microsomal membrane (MM). HeLa cells were incubated for 24 h with various concentrations of VPTLK, and subcellular fractions were analyzed by MS as described in Expeimental Procedures. Each value represents the mean of triplicate samples and standard error.

The amount of VPTLK that penetrates into HeLa cells was measured by using a mass spectrometric [LC-MS/MS (Liquid Chromatography Mass Spectrometer)] method. Isotopically labeled VPTLK [VPTL(^13^C^15^N)K] was added to the cell lysate at a known concentration prior to the peptide extraction steps so that VPTL(^13^C^15^N)K could be used as an internal standard. HeLa cells were cultured in medium containing concentrations of VPTLK ranging from 0.5 μM to 1.6 mM. The amount of VPTLK that entered into HeLa cells increased in a dose-dependent manner (Figure 4a and b). However, it reached an intracellular plateau of approximately 6 μM when the VPTLK concentration in the culture medium was greater than 800 μM. These results suggest that the cell entry of VPTLK is not merely by a simple diffusion mechanism. Interestingly, the cell entry of Fam-VPTLK showed a linear increase up to the maximum concentration examined (1.6 mM) (Figure 4c and d), indicating that Fam-VPTLK and VPTLK use different cell-entry pathways, at least when the extracellular VPTLK concentration is above 800 μM. 

Cells were subjected to subcellular fractionation, and the distribution of VPTLK in each fraction was determined (Figure 4e). VPTLK localized mainly in the cytosol and did not accumulate in the nucleus, nor in the heavy membrane fraction (HM) that contains mitochondria, nor in the endoplasmic reticulum (ER). The cytosolic localization of VPTLK was also observed when fluorescence microscopy was used to detect Fam-VPTLK (Figure 1c), which is consistent with the result of LC-MS/MS analysis of non-labeled VPTLK.

### 2.5. CPP5s have protein-transduction activity

**Figure 5:**
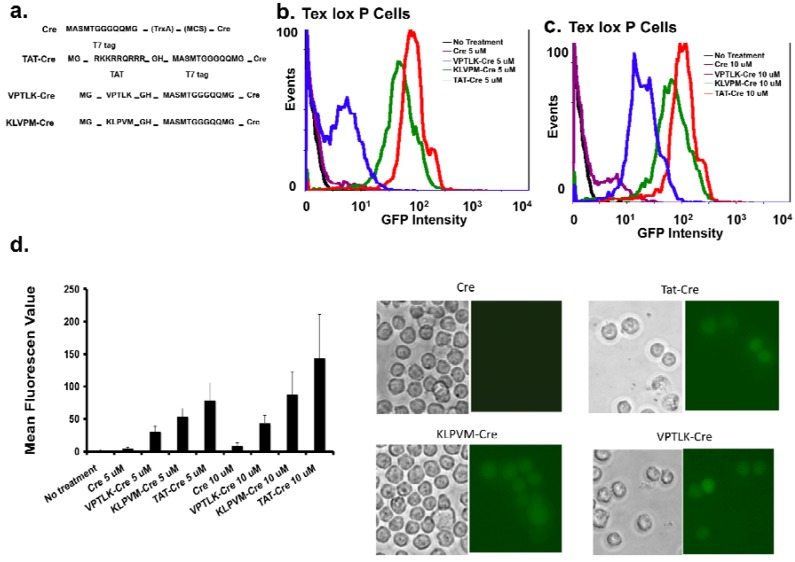
Protein-Transduction activity (PTDs) of VPTLK and KLPVM determined by the delivery of Cre recombinase into cells. *a.* Scheme of Cre protein, TAT-Cre, VPTLK-Cre, and KLPVM-Cre examined in this study. *b-e.* Tex-loxP-EG cells were incubated with purified recombinant Cre, VPTLK-Cre, KLPVM-Cre, and TAT-Cre (5 and 10 μM) for 3 h in serum-free *(b-d)* or 10% serum-containing *(e)* media. After the incubation of cells with CPP-Cre fusion proteins, medium was changed to 10% FCS containing DMEM and cells were culture for additional 45 hrs. The delivery of Cre into Tex-lox P-EG cells was evaluated by GFP expression as previously reported [11]. GFP expression was analyzed 48 h after the incubation of cells with Cre proteins. *b and c.* FACS analysis of GFP expression in cells incubated with 5 μM *(b)* or 10 μM *(c)* Cre and CPP-Cre fusion proteins in serum-free medium. Representative data from three independent experiments are shown. *d.* Mean GFP fluorescence intensity of Tex-loxP-EG cells incubated with 5 or 10 μM Cre recombinant proteins under serum-free conditions. Each bar represents three independent experiments and standard error. *e.* Tex-loxP-EG cells incubated with purified recombinant Cre, VPTLK-Cre, KLPVM-Cre, and TAT-Cre (5 μM) in 10% serum-containing medium for 3 hrs. Images were captured 48 h after the incubation to detect GFP expression (right panel).

To determine the protein-transduction activity of VPTLK and KLPVM, these peptides were added to the N-terminus of Cre DNA recombinase and the resulting CPP5-Cre fusion proteins were added separately to the culture medium of Tex-loxP-EG cells, which are engineered to express GFP through Cre-dependent excision of a chromosomal lox P site. This method was developed by S. Dowdy’s group to determine the protein-transduction activity of TAT peptide [11]. As shown in Figure 5a, the TAT sequence of the TAT-Cre expression plasmid was replaced with VPTLK and KLPVM and these plasmids were used to express recombinant proteins.

First, cells were cultured for 3 h in serum-free medium in the presence of 5 or 10 µM Cre, TAT-Cre, KLPVM-Cre, or VPTLK-Cre. GFP expression was measured 48 h after the incubation with the CPP-Cre proteins. As shown in Figure 5b-d, KLPVM and VPTLK were able to induce GFP expression similar to TAT did, whereas Cre itself did not. In serum-free medium, KLPVM and TAT showed similar levels of protein-transduction activity, and VPTLK showed lower activity than the other CPPs. Second, Cre fusion proteins (5 µM) were added to the culture medium containing serum (10%), and GFP expression was examined 48 h after the treatment (Figure 5 e). All of TAT-Cre, VPTLK-Cre, and KLPVM-Cre were able to induce GFP expression, suggesting that all these CPPs are able to deliver the cargo protein in 10% serum-containing medium. We also sought to examine the addition of 10 μM Cre-fusion proteins to serum-containing medium; however, precipitation of serum proteins occurred and cell viability became very low under these conditions. Probably, the high-salt solubilization buffer (10 mM phosphate and 500 mM NaCl, pH 6.8) of the fusion proteins caused the serum proteins to precipitate.

### 2.6. Discussion

The present study showed that two CPP5s (KLPVM and VPTLK) have protein-transduction activity, as determined by the delivery of Cre protein into cells from the culture medium. Previously, we reported that these CPP5s were able to deliver GFP from the medium into the cell [6]. CPP5 was attached to the N-terminus of Cre (Figure 5 a) and to the C-terminus of GFP [6]. Both cargo proteins (i.e. Cre and GFP) were delivered into the cell. Therefore, CPP5 is able to express protein transduction activity whether at the N- or C-terminus of the cargo protein. In this study, we focused on analyzing VPTLK and KLPVM for their protein-transduction activities, and it is not yet known whether other CPP5s have such capabilities. Further studies examining the protein-transduction activities of other CPP5s are necessary to find the best CPP5s for protein delivery across the plasma membrane.

Cationic peptides such as TAT and poly-arginine have been widely used as a means for protein transduction [10,11,19,35]. As shown in Figure 1, CPP5s have less toxicity than arginine-rich peptide. The low toxicity of CPP5s may be an advantage over TAT in terms of delivery of large amounts of therapeutic cargo molecules into damaged cells to restore their function. Examination of the protein-transduction activity of CPP5s in animal models is clearly the next important experiment to be undertaken.

VPTLK localizes mostly in the cytosol. Since VPTLK binds cytosolic Bax protein [6,22,23], Bax may serve as the cytosolic retention factor for VPTLK. It is also possible that other VPTLK-binding factors exist in the cytosol. Importantly, VPTLK-Cre was able to switch on Cre-inducible GFP gene expression, indicating that VPTLK-Cre was able to reach chromosomal DNA in the nucleus. This means that VPTLK did not interfere with the nuclear-localization signal of Cre protein. Therefore, VPTLK can be utilized for the delivery of nuclear proteins into the cell even though VPTLK itself has a propensity to localize in the cytosol.

By means of a mass spectrometry-based method, we succeeded in performing a quantitative analysis of the cell-penetrating activity of VPTLK. We found that VPTLK reached a concentration of 6 μM in the cytosol when the culture medium contained 800 μM VPTLK (Figure 4 e); however, the intracellular concentration of VPTLK did not increase further even after the concentration of VPTLK in the medium was increased from 800 μM to 1.6 mM. The intracellular VPTLK concentration showed a sigmoid curve in relation to the extracellular VPTLK concentration. This result suggests that simple diffusion is not the mechanism of VPTLK entry into the cell, but that there is a specific cell-entry pathway for VPTLK such as a receptor-mediated mechanism. In contrast, the intracellular concentration of Fam-VPTLK did not reach a plateau, suggesting that Fam-labeled VPTLK and non-labeled VPTLK use different mechanisms to enter the cell, at least at high concentrations (>800 μM) in the culture medium. 

We speculate that Fam helps VPTLK to integrate into the plasma membrane at high concentrations because Fam has high affinity for the plasma membrane, as evidenced by its application to visualize histological structure of the retina [36]. The mechanism of cell entry of CPP5s is still unknown. The present study showed that almost all of the tested inhibitors of pinocytosis and endocytosis were unable to block the cell entry of Fam- or Tamra-labeled CPP5s (Figure 2 and Figure 3). To be noted, cytochalasin D was able to attenuate the cell-penetrating activity of Tamra-labeled CPP5s as well as Tamra-labeled R8 (Figure 2d), though other inhibitors did not. The variation in the effects of inhibitors on the cell-entry activity between Fam- and Tamra-labeled peptides suggests that the fluorescent tag itself may have an influence on the cell-entry activity. Our attempts to determine the mechanism of cell entry by examining the effects of inhibitors did not give a clear answer. The present study, at least, suggests that a cytochalasin D-sensitive endocytic pathway may be involved in the cell-penetrating mechanism of CPP5s. However, since all other inhibitors of endocytosis and pinocytosis were not able to inhibit the cell entry of CPP5, it is possible that mechanisms other than endocytosis and pinocytosis play a major role in the cell-penetrating activity of CPP5s.

As listed in Figure 1a, there are numerous penta-peptides that show cell-penetrating activity when the activity is measured by the cellular intake of Fam-labeled peptides. These peptides share similar composition, i.e. they contain one or two charged hydrophilic amino acids at the N- or C-terminus, and two to three hydrophobic amino acids additionally. This composition may allow CPP5s to possess affinity for the phospholipid membrane and/or a putative receptor(s) expressed in the membrane. Further studies of the mechanism of binding of CPP5s to the plasma membrane will provide clues to elucidate the mechanism of cell entry of CPP5s.

It is intriguing that TAT at higher concentrations induced apoptosis, which is the energy-dependent cellular response involving activation of specific proteases (Caspases) to induce programmed cell death^6^. Excess cationic peptides at the cell surface may trigger an apoptotic signal through an unknown mechanism. Identification of the apoptotic signal may help in the development of technologies to reduce the cytotoxicity of TAT. In our previous study, the fusion peptide of TAT and BIP (Bax-Inhibiting Peptide designed from Ku70) reduced the cytotoxicity of TAT in DAMI (a human megakaryocytic cancer cell line) cells^6^. Since BIP attenuated the toxicity of TAT, Bax may be involved in the TAT-induced apoptosis pathway. The combination of TAT and BIP may be one solution to attenuate the toxic side effects of TAT-dependent drug delivery. 

## 3. Experimental

### 3.1. Peptide synthesis and preparation

The synthesis and preparation of peptides were performed as described previously [6,22,23,29]. The peptides were either synthesized at the Blood Research Institute (Milwaukee, WI, USA), or purchased from Biopeptide Co., Inc. (San Diego, CA, USA) and New England Peptides LLC (Gardner, MA). VPTL^13^C^15^NK was purchased from Sigma-Aldrich (Saint Louis, MO). Dried peptide powders were stored at –80°C. The peptides were dissolved in fresh dimethyl sulfoxide (DMSO; Sigma) at 200 mM in plastic tubes (Costar), and 5 µL of each solution was dispensed to individual 0.5-mL plastic tubes (Costar). These 5-µL aliquots were used as stocks. All tubes were stored at –20°C, and each tube was used only once to minimize freeze-thaw degradation [22,23,29].

### 3.2. Cell culture and transfection

HeLa and CHO cells were cultured in Dulbecco’s modified Eagle’s medium (DMEM) supplemented with 10% fetal bovine serum (FBS). DAMI (a human megakaryocytic cancer cell line) cells were cultured in Iscove’s Modified Dulbecco’s medium (IMDM: Gibco) supplemented with 10% horse serum. Mouse thymoma Tex loxP EG (Tex-loxP-EG) cells, and Jurkat cells were cultured in RPMI medium supplemented with 10% FBS. Mouse Embryonic Fibroblasts (MEFs) were cultured in DMEM medium supplemented with 10% FBS, and sodium pyruvate 1%, nonessential amino acids 1%, and penicillin/streptomycin 1%. The plasmid transfections were performed using Superfect® (Qiagen, Valencia, CA) in accordance with the manufacturer’s instructions.

### 3.3. Apoptosis induction and detection

To induce Bax-mediated apoptosis in HeLa cells, cells were transfected with pEGFP-Bax (1 µg) and apoptosis was determined 24 h after the transfection. Other apoptotic stresses used in other cell lines were staurosporine (STS-100 nM) or etoposide (10 µM) treatment [37]. To detect apoptosis, cells were stained with Hoechst 33258 dye, and the numbers of cells with apoptotic nuclei were counted using fluorescence microscopy. Three-hundred cells were analyzed in triplicate samples. The data presented in the figures show the percentage of apoptosis ± SEM of three independent experiments.

### 3.4. Apoptosis measurements as a biological readout for cell penetration

VPTLK, VPMLK, VPALR, and VPALK are CPP5 peptides that bind and inhibit Bax activation, and are named Bax-Inhibiting Peptides (BIP - Figure 1a). In this study, VPTLK was used as a representative BIP for the confirmation of cell penetration using Bax inhibition as biological readout for the cell entry into mammalian cells. To determine if VPTLK penetrates into mammalian cells that lack a pinocytotic pathway, Chinese Hamster Ovary (CHO A745) cells that lack proteoglycans in the plasma membrane were used [32], and Jurkat cells that have a deficiency in caveolin 1-mediated endocytosis were used [38]. CHO A745 cells, CHO K1 cells (wild type for proteoglycans), or Jurkat cells were pre-incubated with VPLTK for 3 h prior to addition of staurosporine (100 nM). Apoptosis induced by staurosporine treatment was determined at 3 and 6 h after treatment by counting apoptotic nuclei using Hoechst dye nuclear staining. Three-hundred cells were counted in triplicate samples. Figures show the percentage of apoptosis ± standard error of three independent experiments. 

### 3.5. Analysis of cell entry of CPPs

For examination of the cell-penetrating activity of 5,6-carboxyfluorescein (Fam)-labeled CPP5 peptides (Fam-VPMLK, Fam-VPTLK), DAMI cells and HeLa cells (5.0×10^4^ cells/mL) were incubated with different concentrations of Fam-labeled peptides for different time periods. Excess peptides were removed by extensive washing with Hanks’ buffered saline solution (HBSS) as previously reported [6,22,23,29] and cellular entry of Fam-labeled peptides was analyzed using a fluorescence microscope (Nikon; TE200), a confocal scanning laser microscope (Leica; TCS SP2), and a FACS instrument (XL Aria), or a Coulter EPICS® XL-MCR.

### 3.6. Examination of the effect of pinocytosis inhibitors

HeLa cells were incubated with each inhibitor in serum-free DMEM medium for 30 min, followed by incubation for 1 h with Fam- or Tamra-labeled CPP5s (VPTLK or KLPVM) (30 µM). Then, the cells were extensively washed with HBSS and complete DMEM medium prior to the analysis with fluorescence microscopy. The inhibitors used were amiloride (an inhibitor of macropinocytosis, 5 µM), cytochalasin D (an inhibitor of F-actin elongation, resulting in inhibition of macropinocytosis and caveolin-mediated endocytosis, 10 µM), chlorpromazine (an inhibitor of clathrin-mediated endocytosis, 28 µM), nocodazole (an inhibitor of microtubule formation, 2 µM), methyl beta-cyclodextrin (it depletes cholesterol from the plasma membrane, resulting in the inhibition of cell penetration that is dependent on lipid rafts [33], 20 µM), and Filipin III (an inhibitor of macropinocytosis, 5 µM). Fam-labeled TAT (YGRKKRRQRRR, 10 µM), and Fam- or Tamra-labeled poly arginine peptide RRRRRRRR (R_8_, 10 µM) were used as controls to determine the effects of inhibitors on CPPs. To verify the effectiveness of the pinocytosis inhibitors examined, rhodamine-labeled EGF (100 ng/mL – 15 nM), and FITC-labeled transferrin (100 µg/mL – 1.4 µM) were used as positive controls [33], and inhibition of the cell entry of rhodamine-EGF and FITC-transferrin by inhibitors was confirmed (not shown).

### 3.7. Mass spectrometry analysis of CPP5 cell entry (*Figure S1*)

HeLa cells were incubated with various concentrations of VPTLK for 24 h, after which the cells were subjected to an acid wash (pH 5.0) to remove peptides attached to the cell surface, according to the previously reported method [39]. Cells were collected by cell lifters (disposable cell lifter, Fisher cat # 08-773-1). After counting the cells, the cells were pelleted by centrifugation. Then, the cells were resuspended in hypotonic buffer [20 mM HEPES, pH 8.0, 10 mM KCl, 1.5 mM MgCl_2_, 1 mM EDTA, 0.1 mM PMSF (added before use), 250 mM sucrose] supplemented with protease inhibitor cocktail (Sigma 1:100 dilution) and DTT (0.01 mM), and lysed using a Dounce homogenizer. After the cell lysis, acetonitrile and sodium chloride (NaCl) were added to final concentrations of 50% and 500 mM, respectively, to the cell lysate. Acetonitrile and NaCl were added to the samples to enhance the dissociation of VPTLK from other proteins. VPTL(^13^C^15^N)K was used as internal standard and added to the cellular lysate at this point in the procedure. The insoluble fraction was separated by centrifugation at 14,000 rpm for 30 min at 4 °C. The supernatant was saved as the total soluble fraction. Proteins in the sample were denatured by heating the samples to 95 °C for 5 min. Samples were dried, dissolved in 0.1% formic acid, desalted using a C18 column (Vydac), and dried. Dried samples were dissolved in 0.02% trifluoro acetic acid (TFA) prior to LC-MS/MS analysis. The LC-MS/MS system used to quantify the amount of VPTLK was equipped with a Digonex HPLC coupled to a 4000 Q Trap (Sciex, Toronto, ON) mass spectrometer with a turbo ion spray source. TFA-dissolved samples (20 µL) were injected into a reverse-phase C18 column (2.1 × 150 mm, 5 µm, 300 Å, Vydac), equilibrated with 10% acetonitrile/0.1% formic acid in water, and the samples were eluted with the same solvent with a flow of 100 µl/min. The ion spray voltage and ion source probe were set to 5 kV and 200 °C, respectively. Nitrogen was used as the nebulizer and auxiliary gas. Multiple reaction monitoring mode was used to monitor VPTLK and VPTL(^13^C^15^NK). The precursor and fragment ions monitored were *m/z* 458.0 VPTLK and 466.1 VPTL(^13^C^15^NK). The collision energy was 36 eV for analyte and internal standard, and nitrogen was used as a collision gas [40]. The amount of VPTLK in the samples was quantified using the chromatographic peak area ratios of the analyte over the internal standard, assuming identical ionization efficiencies and identical yields of fragment ions for VPTLK and VPTL(^13^C^15^NK), using Analist software 1.4.1 (Sciex, Toronto, ON, Canada) [40]. The analytical method showed linearity between the concentrations 0.1 nM to 10,000 nM.

**Figure S1:**
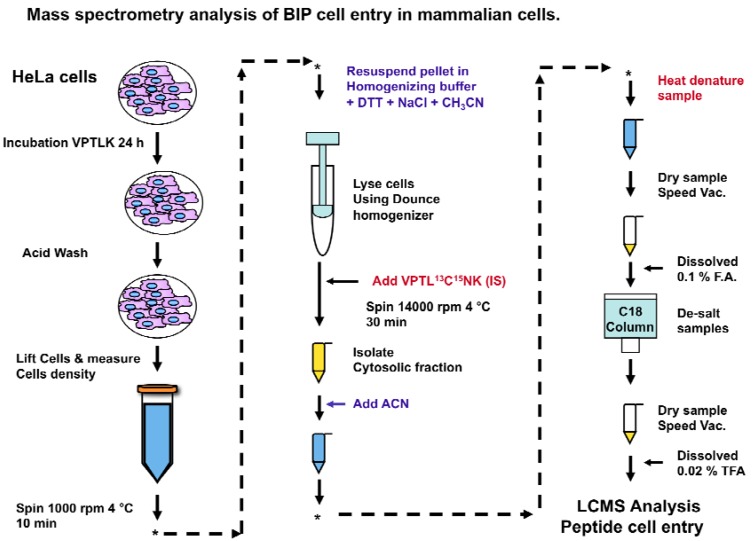
Mass spectrometry scheme. Scheme presenting the critical steps for the mass spectrometric analysis of VPTLK concentration in HeLa cells.

### 3.8. Cellular fractionation

Cells were pelleted at 1,000 rpm for 10 min at 4 °C. Then, the cells were resuspended in hypotonic buffer (20 mM HEPES pH 8.0, 10 mM KCl, 1.5 mM MgCl_2_, 1 mM EDTA, 0.1 mM PMSF, 250 mM sucrose) supplemented with protease inhibitor cocktail (Sigma 1:100 dilution) and DTT (0.01 mM), and the cells were lysed using a Dounce homogenizer. After the lysis, acetonitrile and NaCl were added to the cell lysate at final concentrations of 50% and 500 mM, respectively. Subcellular fractionation was performed as follows: cell lysates were centrifuged at 4000 rpm (Eppendorf microcentrifuge) for 10 min at 4°C. The pellet was used as the nuclear fraction after re-washing the pellet in homogenization buffer. The supernatent was subjected to centrifugation at 14,000 rpm for 30 min at 4°C. The pellet was used as the heavy membrane (HM) fraction. The supernatent was subjected to further centrifugation at 36,000 rpm for 90 min at 4 °C to separate the cytosol (supernatant) and microsomal membrane (MM, pellet) fraction. To all fractions, acetonitrile and NaCl solutions were added. VPTL^13^C^15^NK (25 to 100 nM) was added to each sample to determine the concentration of the non-labeled VPTLK peptide by LC-MS/MS.

### 3.9. Recombinant protein preparation

The cDNA sequences of VPTLK and KLPVM replaced the TAT sequence of pET28.2 TAT-Cre plasmid developed by Dr. Dowdy [11]. Using the human codons from Ku70, the cDNA sequences of the CPP5s VPTLK (CAT GGA TGT GAC TTC) and KLPVM (TTC GAC GGA CAT TAC) were inserted into the plasmid. The restriction enzyme used were NcoI and NdeI. The DNA sequence in the plasmid was otherwise kept as the original ^11^. To generate Cre protein without TAT or CPP5s, Cre cDNA was subcloned into the pET32a+ vector. EcoRI and NotI were used to cut and subclone the Cre cDNA into the pET32a+ vector. After verification of the DNA sequence, *E. coli* BL21 rossetta was transformed by these plasmids. Protein production was induced by IPTG (500 µM). His-tagged VPTLK-Cre, KLPVM-Cre, TAT-Cre and Cre were purified using Ni-column chromatography. Purified recombinant Cre proteins were dissolved in a high-salt buffer [10 mM phosphate, 500 mM NaCl, 5% glycerol, 10 mM beta mercaptoethanol (BME), pH 6.8]. BME and glycerol were added prior to storing the solutions at -80 °C, and after measuring the protein concentration. The frozen stocks were taken from -80 °C and kept on ice until they were added to the Tex *lox* P EG cells as previously reported [11]. 

### 3.10. Determination of protein transduction

Tex *lox* P EG cells were established from thymoma cells of p53-deficient mice, which were genetically engineered to have a *lox* P site flanking the EGFP gene. These cells were used to test the transduction of Cre by VPTLK, KLPVM, and TAT [11]. The Tex-loxP-EG cells (500,000 cells/ml) were incubated with purified recombinant Cre, VPTLK-Cre, KLPVM-Cre, and TAT-Cre (5 and 10 μM) for 3 h in serum-free or 10% serum-containing medium. Then, medium was changed to 10% FCS containing DMEM and cells were culture for additional 45 hrs. Forty-eight hours after the addition of CPP-Cre fusion protein, the transduction of Cre by VPTLK, KLPVM and TAT was determined by the expression of GFP. Expression of GFP was analyzed by fluorescence microscopy and by FACS [11].

## 4. Conclusions

Cell-penetrating penta-peptides (CPP5s) were first developed as Bax-Inhibiting Peptides (BIPs). Ku70, a multifunctional protein regulating DNA repair and apoptosis, is known to bind Bax, a cell death-inducing protein. BIPs were designed based on the Bax-binding domain of Ku70. In addition to BIPs, other CPP5s were designed by mutating the amino acid sequence of BIPs. In this study, VPTLK (one of the BIPs) and KLPVM (a CPP5 without BIP activity) were used to characterize the cell-penetrating activity of CPP5s. VPTLK and KLPVM were able to enter the cell in the presence of various types of inhibitors of pinocytosis and endocytosis. These CPP5s were able to enter proteoglycan-deficient cells. These results suggest that CPP5s may not utilize the known pathways of pinocytosis and endocytosis in exerting their cell-penetrating activity. By means of mass spectrometry, the intracellular concentration of a CPP5 (in this case VPTLK) was measured. As a result, CPP5 reached an intracellular concentration of 25 nM- 6 μM when the cells were cultured in the medium containing 1 μM- 1.6 mM CPP5. Finally, we confirmed that VPTLK and KLPVM have protein-transduction activity. These CPP5s were fused to Cre protein (a DNA recombinase). These fusion proteins were able to induce GFP expression in cells that were genetically engineered to express GFP genes following activation by Cre. Because CPP5s have very low cytotoxicity, they could be utilized as a new tool for drug delivery into cells.

## References

[1] El-Andaloussi S., Holm T., Langel U. (2005). Cell-penetrating peptides: mechanisms and applications. Curr. Pharm. Des..

[2] Murriel C.L., Dowdy S.F. (2006). Influence of protein transduction domains on intracellular delivery of macromolecules. Expert. Opin. Drug Deliv..

[3] Snyder E.L., Dowdy S.F. (2004). Cell penetrating peptides in drug delivery. Pharm. Res.

[4] Zorko M., Langel U. (2005). Cell-penetrating peptides: mechanism and kinetics of cargo delivery. Adv. Drug Deliv. Rev..

[5] Futaki S. (2005). Membrane-permeable arginine-rich peptides and the translocation mechanisms. Adv. Drug Deliv. Rev..

[6] Gomez J.A., Gama V., Yoshida T., Sun W., Hayes P., Leskov K., Boothman D., Matsuyama S. (2007). Bax-inhibiting peptides derived from Ku70 and cell-penetrating pentapeptides. Biochem. Soc. Trans..

[7] Frankel A.D., Pabo C.O. (1988). Cellular uptake of the tat protein from human immunodeficiency virus. Cell.

[8] Green M., Loewenstein P.M. (1988). Autonomous functional domains of chemically synthesized human immunodeficiency virus tat trans-activator protein. Cell.

[9] Cai S.R., Xu G., Becker-Hapak M., Ma M., Dowdy S.F., McLeod H.L. (2006). The kinetics and tissue distribution of protein transduction in mice. Eur. J. Pharm. Sci..

[10] Schwarze S.R., Ho A., Vocero-Akbani A., Dowdy S.F. (1999). *In vivo* protein transduction: delivery of a biologically active protein into the mouse. Science.

[11] Wadia J.S., Stan R.V., Dowdy S.F. (2004). Transducible TAT-HA fusogenic peptide enhances escape of TAT-fusion proteins after lipid raft macropinocytosis. Nat. Med..

[12] Joliot A., Pernelle C., Deagostini-Bazin H., Prochiantz A. (1991). Antennapedia homeobox peptide regulates neural morphogenesis. Proc. Natl. Acad. Sci. USA.

[13] Derossi D., Joliot A.H., Chassaing G., Prochiantz A. (1994). The third helix of the Antennapedia homeodomain translocates through biological membranes. J. Biol. Chem..

[14] Futaki S., Suzuki T., Ohashi W., Yagami T., Tanaka S., Ueda K., Sugiura Y. (2001). Arginine-rich peptides. An abundant source of membrane-permeable peptides having potential as carriers for intracellular protein delivery. J. Biol. Chem..

[15] Futaki S., Goto S., Sugiura Y. (2003). Membrane permeability commonly shared among arginine-rich peptides. J. Mol. Recognit..

[16] Oehlke J., Scheller A., Wiesner B., Krause E., Beyermann M., Klauschenz E., Melzig M., Bienert M. (1998). Cellular uptake of an alpha-helical amphipathic model peptide with the potential to deliver polar compounds into the cell interior non-endocytically. Biochim. Biophys. Acta.

[17] Pooga M., Hallbrink M., Zorko M., Langel U. (1998). Cell penetration by transportan. FASEB J..

[18] Pooga M., Kut C., Kihlmark M., Hallbrink M., Fernaeus S., Raid R., Land T., Hallberg E., Bartfai T., Langel U. (2001). Cellular translocation of proteins by transportan. FASEB J..

[19] Foerg C., Merkle H.P. (2008). On the biomedical promise of cell penetrating peptides: limits versus prospects. J. Pharm. Sci..

[20] Joliot A., Prochiantz A. (2004). Transduction peptides: from technology to physiology. Nat. Cell Biol..

[21] Jones S.W., Christison R., Bundell K., Voyce C.J., Brockbank S.M., Newham P., Lindsay M.A. (2005). Characterisation of cell-penetrating peptide-mediated peptide delivery. Br. J. Pharmacol..

[22] Li Y., Yokota T., Gama V., Yoshida T., Gomez J.A., Ishikawa K., Sasaguri H., Cohen H.Y., Sinclair D.A., Mizusawa H., Matsuyama S. (2007). Bax-inhibiting peptide protects cells from polyglutamine toxicity caused by Ku70 acetylation. Cell Death Differ..

[23] Yoshida T., Tomioka I., Nagahara T., Holyst T., Sawada M., Hayes P., Gama V., Okuno M., Chen Y., Abe Y., Kanouchi T., Sasada H., Wang D., Yokota T., Sato E., Matsuyama S. (2004). Bax-inhibiting peptide derived from mouse and rat Ku70. Biochem. Biophys. Res. Commun..

[24] Downs J.A., Jackson S.P. (2004). A means to a DNA end: the many roles of Ku. Nat. Rev. Mol. Cell Biol..

[25] Gama V., Gomez J.A., Mayo L.D., Jackson M.W., Danielpour D., Song K., Haas A.L., Laughlin M.J., Matsuyama S. (2009). Hdm2 is a ubiquitin ligase of Ku70: Akt promotes cell survival by inhibiting Hdm2-dependent Ku70 destabilization. Cell Death Differ..

[26] Nakase I., Tadokoro A., Kawabata N., Takeuchi T., Katoh H., Hiramoto K., Negishi M., Nomizu M., Sugiura Y., Futaki S. (2007). Interaction of arginine-rich peptides with membrane-associated proteoglycans is crucial for induction of actin organization and macropinocytosis. Biochemistry.

[27] Qin Q., Patil K., Sharma S.C. (2004). The role of Bax-inhibiting peptide in retinal ganglion cell apoptosis after optic nerve transection. Neurosci. Lett..

[28] Tanaka K., Kobayashi N., Gutierrez A.S., Rivas-Carrillo J.D., Navarro-Alvarez N., Chen Y., Narushima M., Miki A., Okitsu T., Noguchi H., Tanaka N. (2006). Prolonged survival of mice with acute liver failure with transplantation of monkey hepatocytes cultured with an antiapoptotic pentapeptide V5. Transplantation.

[29] Gomez J.A., Gama V., Matsuyama S. (2006). Cell-permeable penta-peptides derived from Bax-inhibiting peptide. Cell Penetrating Peptide.

[30] Conner S.D., Schmid S.L. (2003). Regulated portals of entry into the cell. Nature.

[31] Suzuki T., Futaki S., Niwa M., Tanaka S., Ueda K., Sugiura Y. (2002). Possible existence of common internalization mechanisms among arginine-rich peptides. J. Biol. Chem..

[32] Sandgren S., Cheng F., Belting M. (2002). Nuclear targeting of macromolecular polyanions by an HIV-Tat derived peptide. Role for cell-surface proteoglycans. J. Biol. Chem..

[33] Rhee M., Davis P. (2006). Mechanism of uptake of C105Y, a novel cell-penetrating peptide. J. Biol. Chem..

[34] Pujals S., Giralt E. (2008). Proline-rich, amphipathic cell-penetrating peptides. Adv. Drug Deliv. Rev..

[35] Schwarze S.R., Dowdy S.F. (2000). *In vivo* protein transduction: intracellular delivery of biologically active proteins, compounds and DNA. Trends Pharmacol. Sci..

[36] Jordan J.F., Diestelhorst M., Grisanti S., Krieglstein G.K. (2003). Photodynamic modulation of wound healing in glaucoma filtration surgery. Br. J. Ophthalmol..

[37] Jacobsen M.D., Weil M., Raff M.C. (1996). Role of Ced-3/ICE-family proteases in staurosporine-induced programmed cell death. J. Cell Biol..

[38] Colin M., Mailly L., Rogee S., D'Halluin J.C. (2005). Efficient species C HAdV infectivity in plasmocytic cell lines using a clathrin-independent lipid raft/caveola endocytic route. Mol. Ther..

[39] Desai M.P., Labhasetwar V., Amidon G.L., Levy R.J. (1996). Gastrointestinal uptake of biodegradable microparticles: effect of particle size. Pharm. Res..

[40] Hajkova D., Rao K.C., Miyagi M. (2006). pH dependency of the carboxyl oxygen exchange reaction catalyzed by lysyl endopeptidase and trypsin. J. Proteome. Res..

